# Freestanding Graphene Fabric Film for Flexible Infrared Camouflage

**DOI:** 10.1002/advs.202105004

**Published:** 2021-12-16

**Authors:** Guang Cui, Zhe Peng, Xiaoyan Chen, Yi Cheng, Lin Lu, Shubo Cao, Sudong Ji, Guoxin Qu, Lu Zhao, Shaokai Wang, Shida Wang, Yizhen Li, Haina Ci, Maoyuan Li, Zhongfan Liu

**Affiliations:** ^1^ Beijing System Design Institute of Mechanical‐Electrical Engineering Beijing 100871 P. R. China; ^2^ Center for Nanochemistry (CNC) Beijing Science and Engineering Center for Nanocarbons College of Chemistry and Molecular Engineering Peking University Beijing 100871 P. R. China; ^3^ Beijing Graphene Institute (BGI) Beijing 100095 P. R. China; ^4^ Shandong Academy of Agricultural Sciences Jinan 250100 P. R. China; ^5^ Ningbo Innovation Research Institute Beihang University Ningbo 315800 China; ^6^ College of Energy Soochow Institute for Energy and Materials InnovationS (SIEMIS) Jiangsu Provincial Key Laboratory for Advanced Carbon Materials and Wearable Energy Technologies Soochow University Suzhou 215006 P. R. China; ^7^ School of Mechanical and Electrical Engineering Qingdao University of Science and Technology Qingdao 266061 P. R. China

**Keywords:** flexible devices, freestanding, graphene fabric film, infrared camouflage, rewetting

## Abstract

Graphene films, fabricated by chemical vapor deposition (CVD) method, have exhibited superiorities in high crystallinity, thickness controllability, and large‐scale uniformity. However, most synthesized graphene films are substrate‐dependent, and usually fragile for practical application. Herein, a freestanding graphene film is prepared based on the CVD route. By using the etchable fabric substrate, a large‐scale papyraceous freestanding graphene fabric film (FS‐GFF) is obtained. The electrical conductivity of FS‐GFF can be modulated from 50 to 2800 Ω sq^−1^ by tailoring the graphene layer thickness. Moreover, the FS‐GFF can be further attached to various shaped objects by a simple rewetting manipulation with negligible changes of electric conductivity. Based on the advanced fabric structure, excellent electrical property, and high infrared emissivity, the FS‐GFF is thus assembled into a flexible device with tunable infrared emissivity, which can achieve the adaptive camouflage ability in complicated backgrounds. This work provides an infusive insight into the fabrication of large‐scale freestanding graphene fabric films, while promoting the exploration on the flexible infrared camouflage textiles.

## Graphical abstract

A freestanding graphene film is reported by etching graphene‐coated substrate with fabric structure. Moreover, the large‐scale papyraceous freestanding graphene fabric film (FS‐GFF) can be further attached to various shaped substrates and showing a superior electrical conductivity. Meanwhile, the FS‐GFF based infrared camouflage flexible textile device exhibits superior infrared camouflage performance.

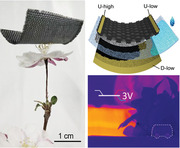

## 1 Introduction

Graphene film (GF) with high mechanical flexibility, superior electrical conductivity, and high specific surface area shows great potentials in supercapacitors,^[^ ^1^, ^2^, ^3^, ^4^ ^]^ gas sensors,^[^ ^5^, ^6^ ^]^ hydrogen storage,^[^ ^7^, ^8^ ^]^ electrodes,^[^ ^9^ ^]^ and field emission displays.^[^ ^10^, ^11^ ^]^ The synthetic strategies of GF can be mainly divided into two routes: 1) top‐down synthesis from the assembly of graphene flakes and its derivative;^[^ ^12^, ^13^, ^14^, ^15^ ^]^ this method enables the GF production with relatively low cost, but suffers from the dilemma of graphene thickness uncontrollability and tedious fabrication procedures; 2) bottom‐up synthesis from carbon source molecule by chemical vapor deposition (CVD) method. The as‐fabricated graphene film owns merits of high quality and high uniformity.^[^ ^16^, ^17^, ^18^, ^19^ ^]^ But the tight integration between graphene film and substrate limits further structure design, while the inevitable tedious transfer process could also cause defects and degrade the properties of graphene.^[^ ^20^, ^21^, ^22^, ^23^, ^24^, ^25^ ^]^

Herein, by virtue of our graphene growth experience on SiO_2_ fiber by CVD method,^[^ ^26^, ^27^, ^28^ ^]^ a flexible papyraceous freestanding graphene fabric film (FS‐GFF) was designed. In case of using nickel/copper fiber as growth substrate, the large diameter (≈100µm) of metal fiber and the loose structure of common metal mesh may cause a degradation of its original fabric structure and make it unable to be freestanding.^[^ ^29^, ^30^, ^31^ ^]^ In this work, the dense structure of SiO_2_ fiber with a small diameter of 6 µm ensured that the FS‐GFF can maintain its original fabric structure after the SiO_2_ fiber was etched. Furthermore, a large‐scale FS‐GFF can be obtained based on the size of SiO_2_ fabric. Moreover, the flexible FS‐GFF can be attached to various shaped objects by a very simple rewetting process.

Based on this novel FS‐GFF, a unique adjustable infrared camouflage (AIC) flexible textile device was fabricated. Benefiting from the substrate‐independent character of the FS‐GFF material, the AIC device can be easily transferred onto any shaped target and operated conveniently compared with other reported works on AIC devices.^[^ ^32^, ^33^, ^34^ ^]^

The infrared emissivity the FS‐GFF‐based AIC device can be modulated from 0.79 to 0.68 by applying a low input voltage of 5 V. Based on a specially designed multielectrode structure, this unique AIC textile device shows excellent infrared camouflage ability under complicated infrared backgrounds.

## 2 Results and Discussion

**Figure 1:**
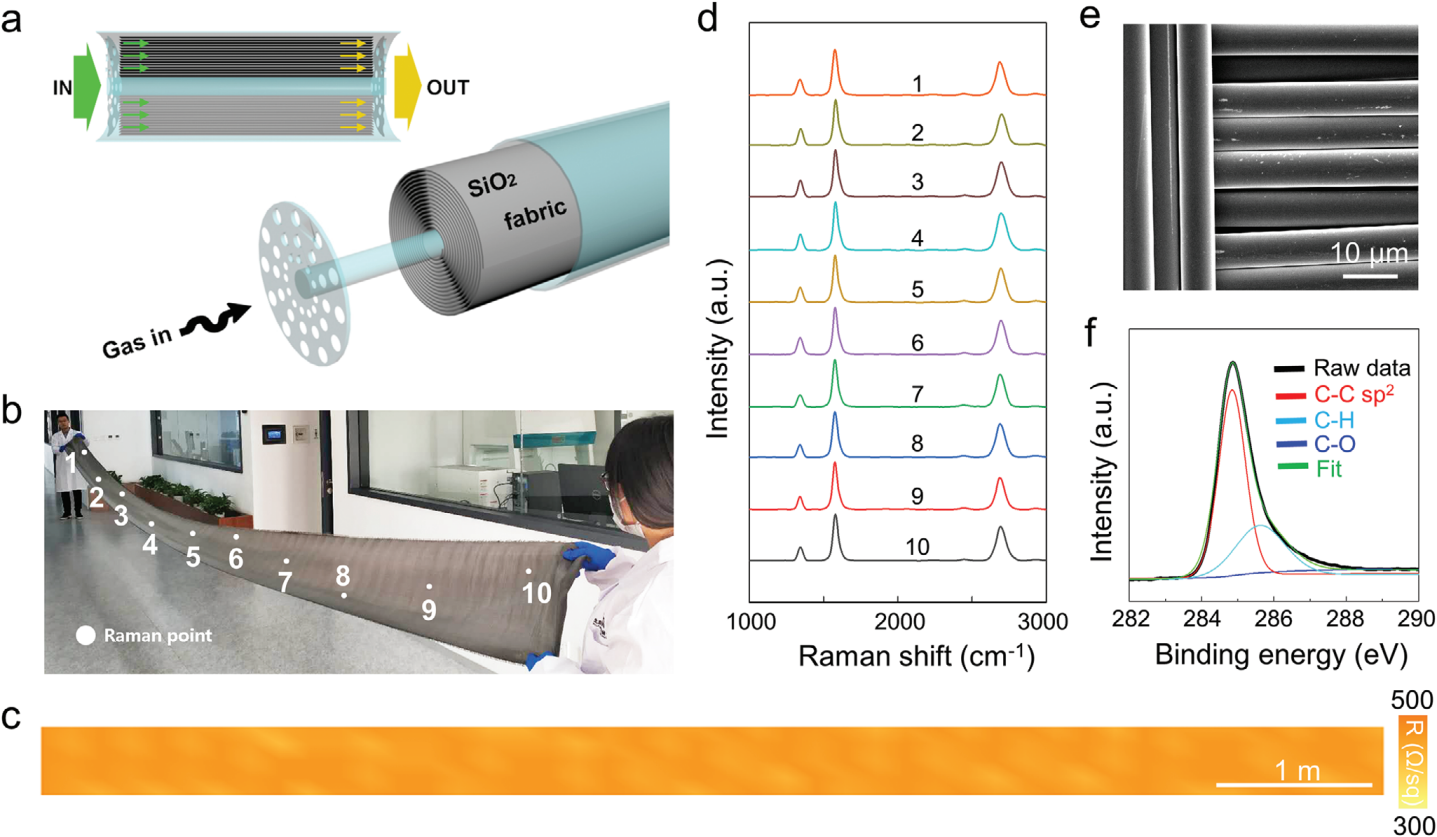
Fabrication of graphene@SiO_2_ fabric (G@SF) by the low‐pressure CVD (LPCVD) method. a) Schematic of the experimental design. 800 cm × 40 cm SiO_2_ fabric was rolled in the CVD chamber. b) 800 cm × 40 cm sized G@SF. c) Sheet resistance mapping of the G@SF. d) Raman spectra of G@SF. e) SEM image of the as‐fabricated G@SF. f) C 1s XPS spectrum of graphene layers.

A roll of SiO_2_ fabric with size about 800 cm × 40 cm (≈320 in. along diagonal direction) was chosen as the flexible substrate for graphene growth (**Figure** **1**a). The SiO_2_ fabric rolled up on quartz holder was plugged into the 6 in. diameter CVD reaction chamber under the low‐pressure CVD (LPCVD) condition. Since the concentration of active carbon species decreased along the gas flow direction, thickness of the graphene film was gradually reduced, resulting in a nonuniform graphene@SiO_2_ fabric (G@SF) sample with lower sheet resistance near the gas‐in direction and higher sheet resistance near the gas‐out direction (Figure S1, Supporting Information). In order to improve the uniformity of the as‐obtained G@SF, an inverted LPCVD process was employed to ensure the homogeneous coverage of graphene film on the SiO_2_ fabric (Figure S2, Supporting Information). Finally, the 800 cm × 40 cm G@SF sample with uniform contrast was obtained, as shown in Figure 1b. The corresponding sheet resistance mapping image indicates its macroscopic homogeneity with average value of ≈400 Ω sq^−1^ (Figure 1c). In addition, the thickness of the graphene films can be modulated by regulating the CVD growth time. With increasing the growth time from 2 to 10 h, thickness of the graphene films can be modulated from 2 to 90 nm, and the corresponded sheet resistance can therefore be tuned from ≈2800 to ≈50 Ω sq^−1^ (Figure S3, Supporting Information).

Raman spectra of the G@SF were collected along the rolling direction from positions marked in Figure 1b. The consistent Raman signals with sharp G peaks and high intensity ratio of G peak and D peak (*I*_G_/*I*_D_) (≈3.4) indicate the large‐scale uniformity and high quality of the as‐fabricated graphene film (Figure 1d). The representative scanning electron microscopy (SEM) morphology of the G@SF confirms its smooth and integral microstructure after LPCVD growth of graphene (Figure 1e). C 1s peak at 284.4 eV with sp^2^‐carbon dominated indicates the high purity of G@SF from the X‐ray photoelectron spectroscopy (XPS) characterization in Figure 1f.

**Figure 2:**
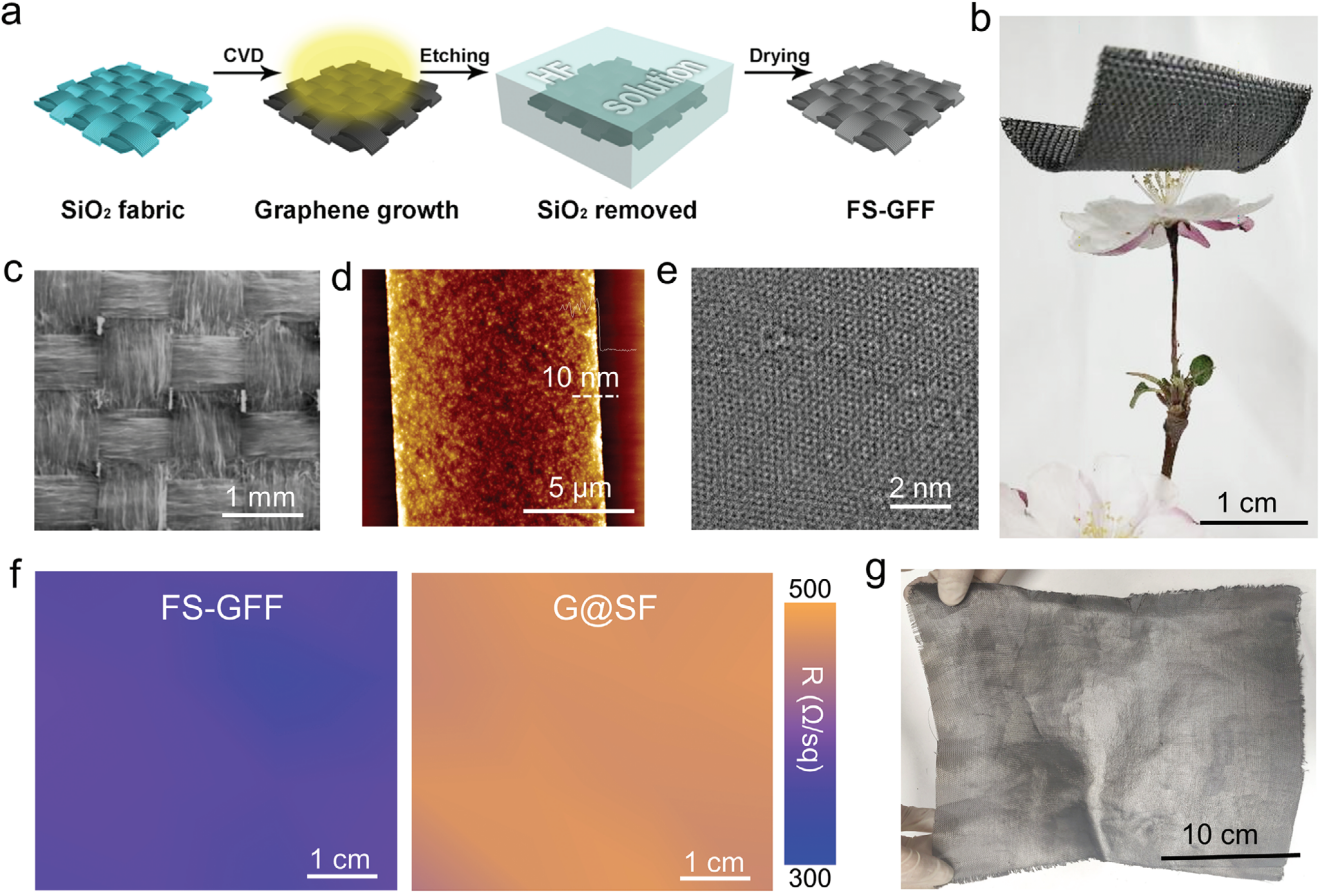
Fabrication of freestanding graphene fabric film (FS‐GFF). a) Schematic of the etching and drying process. b) The as‐fabricated ultralight FS‐GFF. c) SEM image of FS‐GFF with a fabric structure. d,e) atomic force microscope (AFM) (d) and TEM (e) characterizations of a single graphene ribbon in FS‐GFF. f) Sheet resistance mapping of G@SF (left) and the corresponding FS‐GFF (right). g) Photograph of a large‐scale FS‐GFF.

The FS‐GFF was obtained by etching and subsequent drying process, as illustrated in **Figure** **2**a. After etching the core SiO_2_ fiber substrate in hydrofluoric acid solution, the extremely soft and wet graphene film was thus obtained floating on water (Figure S4, Supporting Information). Eventually by a simple collection and drying process, the freestanding graphene film was successfully prepared. Density of the FS‐GFF was measured as ≈0.02 g cm^−3^ (Figure 2b), exhibiting a good performance of lightweight (see Video S1 in the Supporting Information). The as‐fabricated FS‐GFF still maintained its original fabric structure consisting of thousands of graphene fibers, as shown in Figure 2c. Meanwhile, the individual graphene fiber collapses into ≈9 µm width graphene ribbon after the etching process (Figure 2d and Figure S5a (Supporting Information)). In addition, the distinct Moire fringe observed in the transmission electron microscopy (TEM) image evinces the high crystallinity of graphene ribbon (Figure 2e).

Furthermore, the FS‐GFF shows superior electrical conductivity compared with that of G@SF (Figure 2f), which can be attributed to the ampliative contact area among individual graphene ribbons (Figure S5b, Supporting Information). Based on the unique fabric structure, the papyraceous FS‐GFF also shows good flexibility. By bending the sample for 12 cycles with a radius of 3 cm, the negligible change in sheet resistance of the FS‐GFF was observed (Figure S6, Supporting Information). Benefiting from our experimental design, a large‐scale FS‐GFF with A4 (30 cm × 20 cm) size can be obtained (Figure 2g). The sample size can be further scaled up under an appropriate growth device and operation, confirming its application potential in the engineering area.

**Figure 3:**
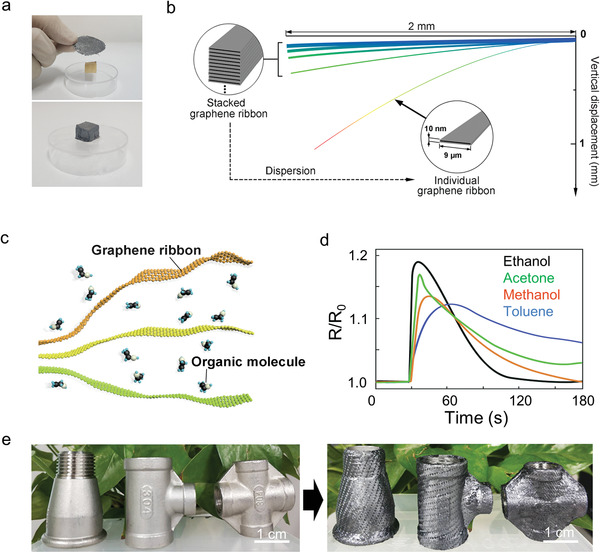
Rewetting process and resoft mechanism of the FS‐GFF. a) FS‐GFF was transferred onto a shaped object. b) Finite element analysis of the resoft process. c) Schematic of graphene ribbon dispersion in organic solution. d) Resistance variation of the FS‐GFF rewet by different organic solutions. e) FS‐GFF on various shaped objects.

In the experimental process, we found that the as‐fabricated papyraceous FS‐GFF can become soft again after a simple rewetting manipulation, being conveniently transformed onto various shapes. At the beginning, the tailored FS‐GFF was subjected onto a target object, as shown in the top picture of **Figure** **3**a. Next, tiny amount of ethanol solution as wetting agent was sprayed on the FS‐GFF, making the FS‐GFF soft again. Spontaneously, the soft FS‐GFF can be attached to the shaped object firmly, replicating perfectly the shape of the target object (bottom picture of Figure 3a). The unique resoft feature of the FS‐GFF offers graphene films to further expand application scenarios.

This resoft phenomenon was mainly caused by the dispersion of stacked graphene ribbons into monolayer ones. A finite element analysis was conducted to elaborate the resoft process. As shown in Figure 3b, the thickest stacked multilayer graphene ribbons (20 layers) only tend to bend slightly under gravity. But as the stacked numbers of graphene ribbons decreased from 20 layers to monolayer, the deformation can reach to as large as ≈1 mm for a 2 mm long graphene ribbon, which can make the FS‐GFF soft again.

Due to the good wettability between graphene and organic solution,^[^ ^35^, ^36^, ^37^ ^]^ the organic molecules can permeate into the interspaces among graphene ribbons and drive the stacked graphene ribbons into individual ones (Figure 3c), which was confirmed by the increase of sheet resistance of the FS‐GFF. The electrical conductivity of FS‐GFF rewet by four kinds of organic solvent at fixed amount (≈1 µL) was measured, respectively (Figure 3d and Figure S7 (Supporting Information)). It can be noticed that all samples showed a significant ≈20% increase of sheet resistance, which verified the feasibility of dispersion process of graphene ribbons.

This phenomenon also shows the potential of FS‐GFF to become soft and achieve the shape transformation easily. Following a drying treatment, the soft FS‐GFF will become freestanding again. This resoft and drying cycle can be repeated for multiple times with no obvious variations in the sheet resistance (Figure S8, Supporting Information), demonstrating its high stability and reproducibility. With the aid of the resoft ability, the FS‐GFF can be anchored onto any shaped arbitrary objects intactly (Figure 3e).

Furthermore, based on the high infrared emissivity of normal graphene film,^[^ ^38^ ^]^ the emissivity of FS‐GFF was tested as 0.79, which is close to the infrared emissivity of natural backgrounds (Figure S9, Supporting Information). Hence, when the FS‐GFF was attached onto arbitrary objects with low infrared emissivity, the substrates with distinct shapes could be concealed into the natural background under the detection of infrared camera (Figure S10, Supporting Information), showing a promising prospect in the infrared device.

**Figure 4:**
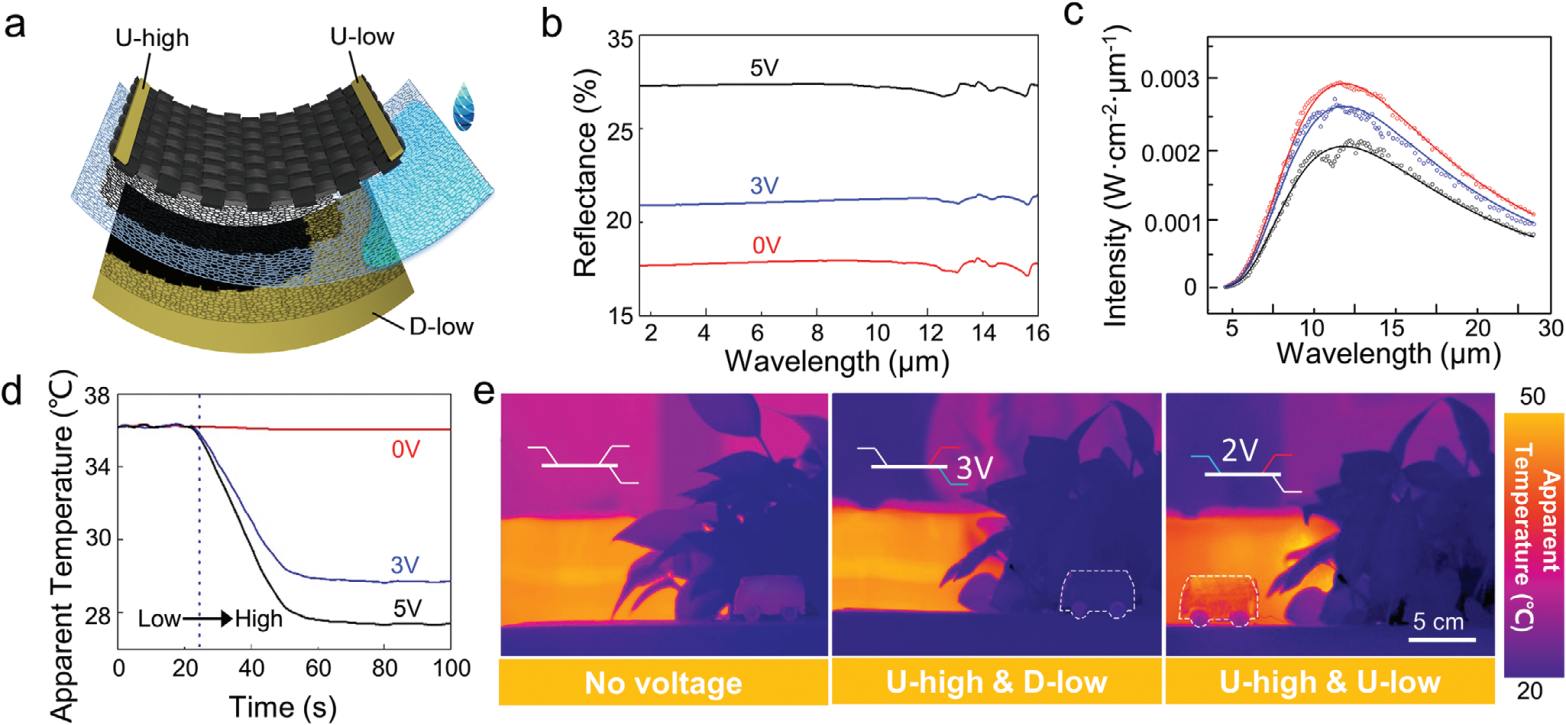
Adjustable infrared camouflage (AIC) flexible textile based on FS‐GFF. a) Schematic of the (AIC) flexible textile device. b,c) Reflectance (b) and infrared emission spectra (c) of the AIC textile device by applying different voltages. d) Apparent temperature change of the AIC textile device by different voltages. e) Infrared camouflage ability of the AIC textile device under complicated environment.

Inspired by the unique infrared radiation performance of graphene materials,^[^ ^38^ ^]^ and considering the high conductivity, and favorable maneuverability of the FS‐GFF, the adjustable infrared camouflage textile device was assembled based on FS‐GFF (**Figure** **4**a). The AIC flexible textile device was composed of the top layer FS‐GFF, middle layer fabric separator, and the back electrode, with 1‐butyl‐3‐methylimidazolium hexafluorophosphate (BMIMPF_6_) as the ionic liquid electrolyte. By applying voltage between the FS‐GFF (U‐high) and the back electrode (D‐low), the ionic motion was induced. When increasing the applied voltage, the infrared reflectance (*R*_infrared_) of the as‐fabricated AIC flexible textile device increases due to the increase of Fermi energy and the optical conductance of FS‐GFF, which is relevant to the intercalation of ions (Figure S11, Supporting Information).^[^ ^39^, ^40^, ^41^ ^]^

According to the Kirchhoff's law, the infrared emissivity of a certain material equals to its absorbance (*A*). Since the FS‐GFF is impermeability to the infrared light with a transmittance (*T*) of zero, the infrared emissivity (*ε*) can be calculated by the equation of *ε* = 1 − *R*_infrared_. Therefore, the infrared reflectance of the AIC device in the infrared atmospheric window can be consequently improved through increasing the applied voltage (Figure 4b). The infrared reflectance of the AIC device is ≈21% without input voltage. When the applied voltage was increased to 5 V, the infrared reflectance can be enhanced to ≈32%. Correspondingly, the infrared emissivity can be modulated from 0.79 to 0.68.

The infrared emission spectra of FS‐GFF at different voltages were also tested, as shown in Figure 4c. The theoretical emission curves of gray body were fitted according to the Planck formula: $E\left(\lambda ,\;T\right)\;=\;(2\pi \varepsilon hc^{2})/(\lambda^{5}(\exp\left(\frac{\mathrm{hc}}{\lambda k_{B}T}\right)\;-\;1))$, where *E*(*λ*, *T*), *ε*, *T*, and *λ* are spectral radiation power of gray body, emissivity, temperature, and wavelength, respectively; *h*, *c*, and *k*_B_ are the Planck constant, speed of light, and the Boltzmann constant, respectively, which shows that the FS‐GFF can be regarded as a representative gray‐body radiation. Based on this adjustable emissivity, the flexible device was attached onto a human finger (Figure S12, Supporting Information). When applying a proper voltage (≈3 V), the infrared emissivity of the AIC device can be decreased, performing a lower temperature than the actual temperature of human finger. Then, the part covered by the AIC device can be concealed in the surrounding environment. The temporal response of the AIC device at different voltages can be recorded by the apparent temperature changes, as illustrated in Figure 4d.

To confirm the infrared camouflage ability of the as‐prepared AIC flexible textile device in complicated substrate and environment, a vehicle model under various infrared background was explored (the assembly details have been supplemented in Figure S13 in the Supporting Information). For simulating the infrared characteristic of actual vehicle, the model was heated to ≈30 °C in an oven previously to simulate the temperature increase caused by passengers and vehicle engine. Under the natural background such as plant, the vehicle model with relative high temperature can be identified easily by the contrast of infrared image (Figure 4e left). By applying a voltage of ≈3 V between U‐high and D‐low electrodes on the AIC device, the apparent temperature of vehicle model was suppressed by the decrease of emissivity. Eventually, the infrared camouflage of the vehicle model could be realized (Figure 4e middle).

To fulfill the requirements of the applications in complicated infrared background, an extra U‐low electrode was applied to the AIC device, as shown in Figure 4a. Based on the good electrothermal performance of graphene fabric,^[^ ^26^, ^42^ ^]^ temperature of the FS‐GFF layer on top of the device was increased, when applying ≈2 V voltage between the U‐high electrode and U‐low electrode on the FS‐GFF layer. So, the vehicle can be concealed into artificial environment with higher temperature (Figure 4e right and Video S2 (Supporting Information)). The as‐fabricated AIC device can control its heat characteristics based on the change of surrounding environment and shield the target from infrared detection, which confirm its high efficiency and adaptability in infrared camouflage field.

## 3 Conclusion

In this work, we prepared a novel FS‐GFF with the tunable electrical conductivity and infrared emissivity by the virtue of CVD method followed by a substrate‐etched process. This growth‐etching process can be further adopted to other nanomaterial assemblies with unique structure and properties, which would be ponderable to broaden the application range of CVD‐based nanomaterials. The thus‐obtained FS‐GFF can be attached to various shaped objects by a simple rewetting process. Based on its flexibility, lightweight, well conductivity, and high infrared emissivity characteristics, the FS‐GFF shows excellent potential as infrared camouflage on any shaped objects. The infrared emissivity of the fabricated AIC flexible device can be modulated from 0.79 to 0.68 by applying a low input voltage of 5 V. By selectively applying voltages, the FS‐GFF textile shows high adaptability in complicated infrared background. Our work features an effective route to prepare the freestanding graphene fabric film toward the deep exploration of graphene film on the application of infrared camouflage.

## 4 Experimental Section

### LPCVD Growth of G@SF

A roll of SiO_2_ fabric about 800 cm × 40 cm was loaded into the 6.0 in. quartz tube of a three‐zone high‐temperature furnace (Lindberg/Blue). Typical growth conditions were 200 sccm of Ar, 100 sccm of H_2_, and 20 sccm of CH_4_ at 1050 °C. The thickness of graphene could be modulated by tailoring the growth time from 2 to 10 h.

### Etching and Drying Process

G@SF was diffused in hydrofluoric acid solution (20 wt%) for hours to remove the SiO_2_ fabric. Afterward, the G@SF collapsed into graphene ribbons and then was washed by deionized water and ethanol to remove the residual chemical reagents. Then, the soft graphene fabric film was transferred to pretreated filter paper. Surface of the filter paper was swept by nitrogen for 30 min and the FS‐GFF was then separated from the filter paper.

### Rewetting Process

First, a piece of FS‐GFF larger than the surface area of the target object was tailored. Second, surface of the target object could be wiped by proper organic solvent. Then, the FS‐GFF was layered on the top of the target object and tiny amount of proper organic solvent was splashed onto the FS‐GFF by nebulizer.

### Simulation

The finite element analysis was conducted to further elaborate the dispersion process. Basic unit of a single graphene ribbon was 20 nm × 9 µm × 2 mm, modulus of elasticity was set as 1.0 TPa, and the number of stacked layers was chosen as 20, 15, 10, 5, and 1.

### Fabrication of the AIC Device on Finger

The fabric separator layer (cellulose paper) was adhered to the electrode (sputtered gold). The other electrode was fixed on the fabric separator layer by conducting resin. Then, the FS‐GFF was attached to the surface of the fabric separator layer by rewetting process, forming the complete device. After the flexible AIC device was transferred to human finger, the ionic liquid electrolyte (BMIMPF_6_) was applied onto the fabric separator layer which acted as both the separator and ionic conductive layer.

### Fabrication of Infrared Camouflage on the Vehicle Model

Cellulose paper was used as separator layer. Sputtered gold was shaped to the back side of the separator layer as D‐low electrode. After the U‐high and U‐low electrodes were fixed, the separator layer was adhered on the vehicle model. By the rewetting process, the FS‐GFF was attached to the top of separator layer and ionic liquid electrolyte was applied onto the fabric separator layer.

### Characterization

The samples were characterized by Raman spectroscopy (Horiba, LabRAM HR‐800, 532 nm laser excitation, 100× objective lens), SEM (Hitachi S‐4800, operating at 1 kV), XPS (Kratos Analytical Axis‐Ultra spectrometer using a monochromatic Al K*α* X‐ray source), optical microscopy (Olympus DX51), Keithley 2001 multimeter (Tektronix, Inc.), and Fourier transform infrared (Nicolet 6700) spectrometer equipped with integrating sphere.

## Conflict of Interest

The authors declare no conflict of interest.

## Supporting information

Supporting Information

Supplemental Video 1

Supplemental Video 2

## Data Availability

Research data are not shared.
