# Supplementary material for: Freestanding Graphene Fabric Film for Flexible Infrared Camouflage
Source: Adv Sci (Weinh). 2021 Dec 16;9(5):2105004. doi: 10.1002/advs.202105004 (PMC8844486; doi:10.1002/advs.202105004)
Supplement: Supplementary file 1 — Supporting Information [file ADVS-9-2105004-s001.pdf]

ADVANCED SCIENCE
Open Access

Supporting Information

for *Adv. Sci.*, DOI: 10.1002/advs.202105004

Free-standing Graphene Fabric Film for Flexible Infrared Camouflage

Guang Cui, Zhe Peng, Xiaoyan Chen, Yi Cheng, Lin Lu, Shubo Cao, Sudong Ji, Guoxin Qu, Lu Zhao, Shaokai Wang, Shida Wang, Yizhen Li, Haina Ci, Maoyuan Li* and Zhongfan Liu**

Supporting Information

Free-standing Graphene Fabric Film for Flexible Infrared Camouflage

Guang Cui, Zhe Peng, Xiaoyan Chen, Yi Cheng, Lin Lu, Shubo Cao, Sudong Ji, Guoxin Qu, Lu Zhao, Shaokai Wang, Shida Wang, Yizhen Li, Haina Ci, Maoyuan Li* and Zhongfan Liu**

The supporting information includes:

Figure S1-13

Video S1-2

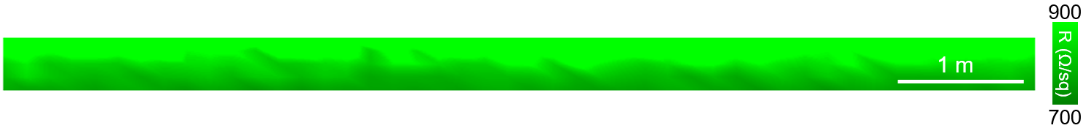

Figure S1. Sheet resistance of the G@SF after the first time LPCVD process.

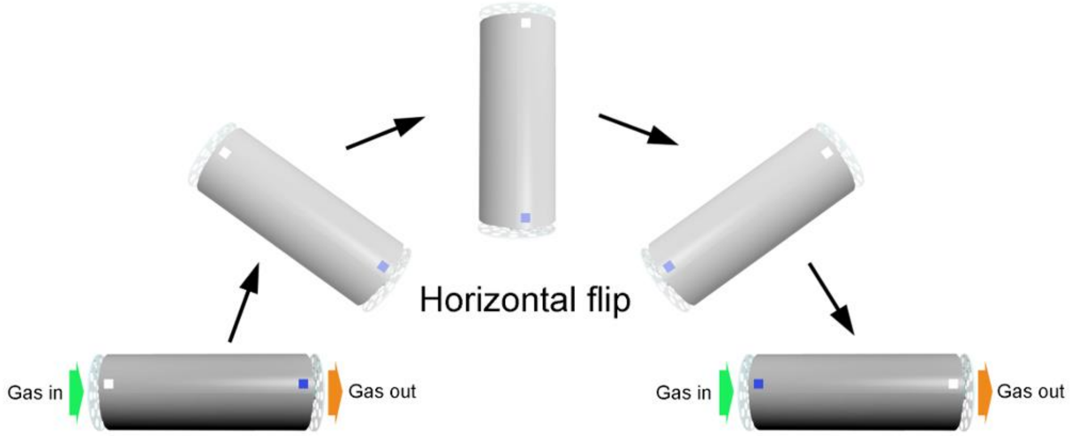

Figure S2. Inverted LPCVD route for the fabrication of uniform G@SF. When the first-round LPCVD process finished, the rolled fabric was cooled down to room temperature. Afterwards, the rolled fabric column was pulled out and turned 180° horizontally. The gas-in side of the fabric column (white marker) was changed into the gas-out position. Then the inverted fabric column was pushed into the CVD chamber for the second-round LPCVD process.

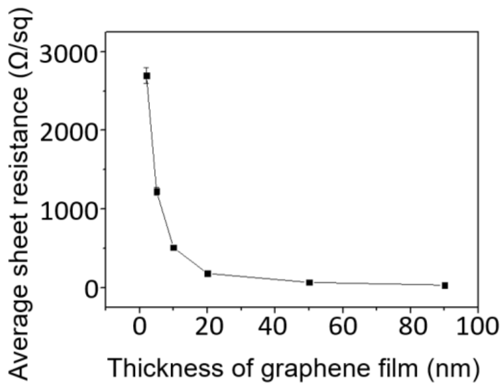

Figure S3. Sheet resistance of the G@SF with different graphene film thickness.

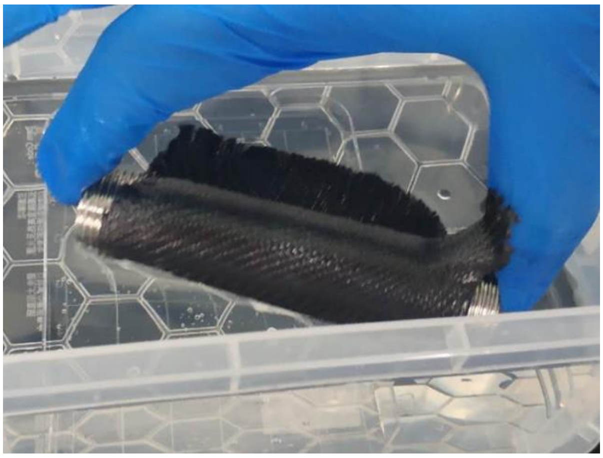

Figure S4. Photo of the soft wet graphene fabric film on water after the SiO_2 substrate was etched.

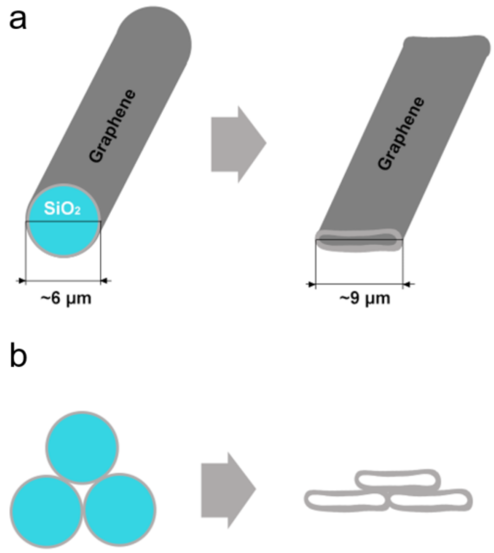

Figure S5. (a) The schematic illustration of a graphene fiber collapsing into a graphene ribbon after the SiO_2 substrate was etched. (b) Corresponded sectional diagram.

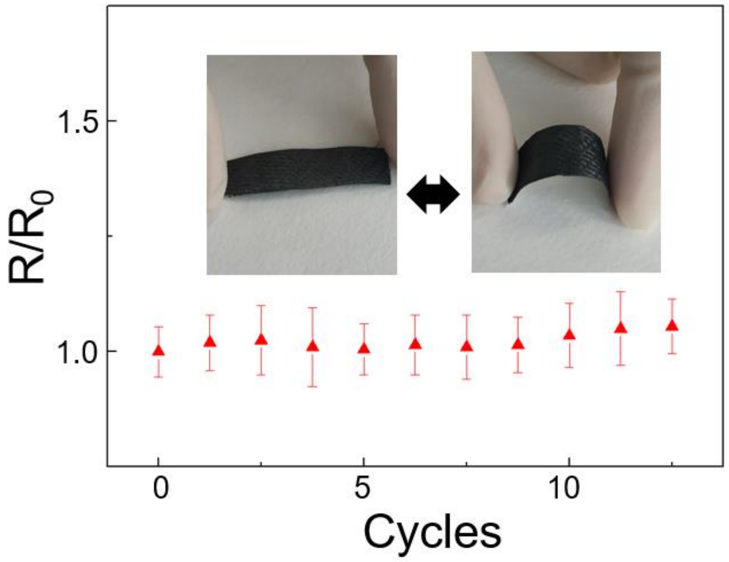

Figure S6. Bending test of FS-GFF with a bending radius of 3 cm for 12 cycles.

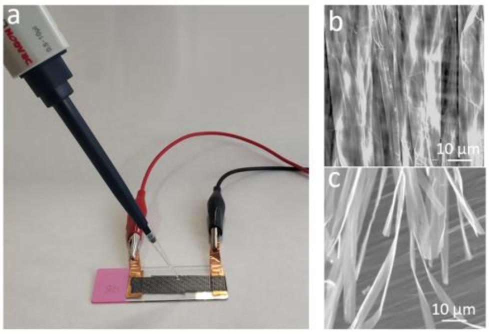

Figure S7. (a) Measurement of the electrical conductivity of FS-GFF rewet by various organic solutions (1μL). (b-c) SEM images of compact graphene ribbons (b) and dispersive graphene ribbons (c).

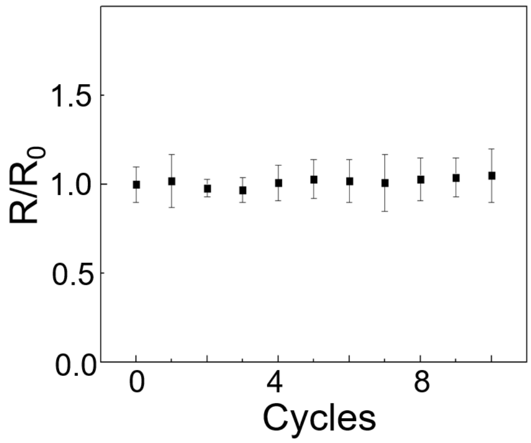

Figure S8. Sheet resistance of the FS-GFF under multiple rewetting and drying cycles.

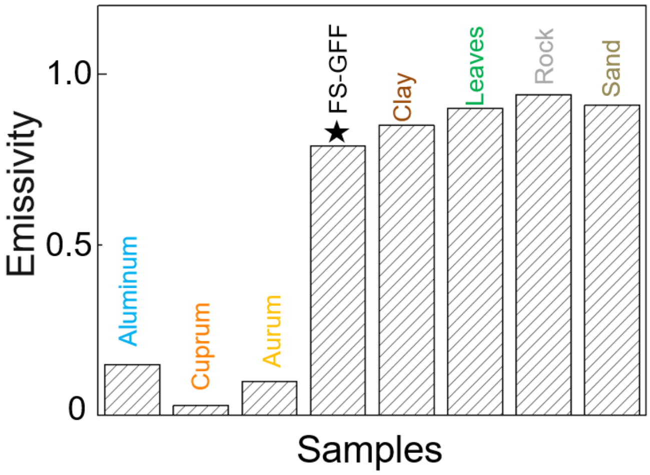

Figure S9. Infrared emissivity of FS-GFF and other natural materials.

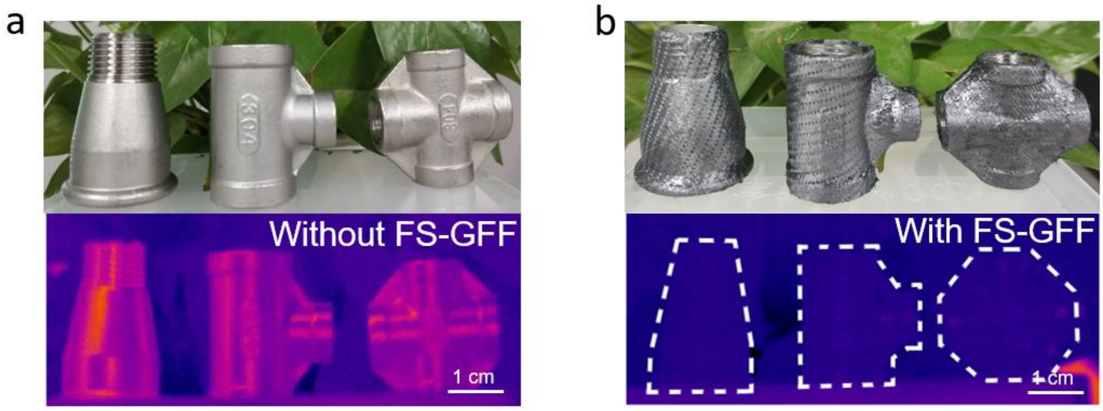

Figure S10. Low emissivity shaped objects with/without FS-GFF and their images under infrared camera.

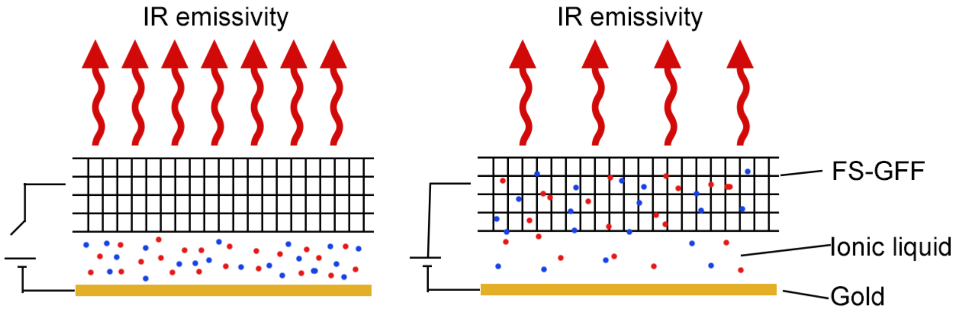

Figure S11. Scheme for the infrared camouflage mechanism of the FS-GFF device. Under a voltage bias, the ionic liquid intercalates into the graphene layers and dopes them. As a result of doping, the charge density of graphene increases and the Fermi-level shifts to higher energies. Thus, the adsorption of photons would be strongly suppressed, leading to a weaker infrared light absorption and emission of the FS-GFF electrode.

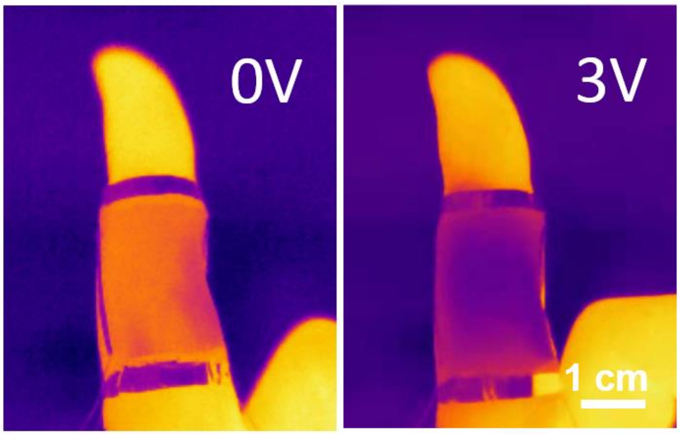

Figure S12. Infrared camouflage ability of the AIC textile device on human finger.

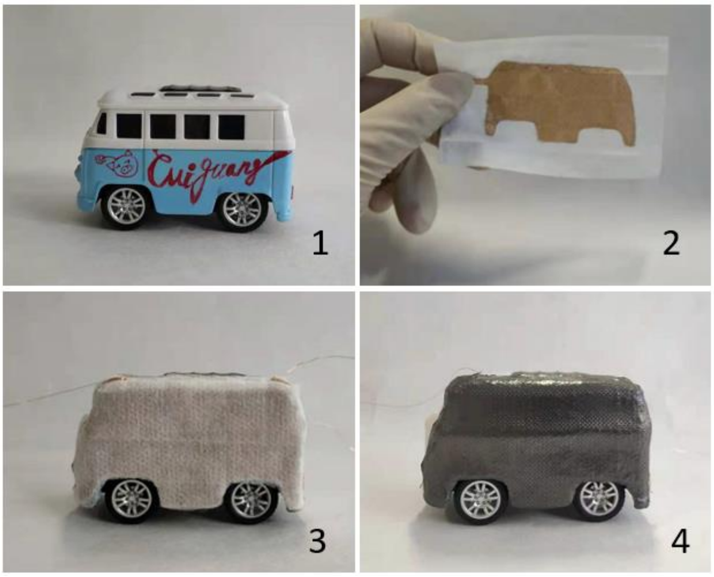

Figure S13. Fabrication of infrared camouflage on vehicle model. Cellulose paper was used as a spacer. Sputtered gold was adhered to the back side of the separator layer as D-low electrode (2). After the gold electrode were fixed, the un-finished textile was adhered on the vehicle model and two copper electrodes was fixed on the front side of the separator layer as U-high and U-low electrodes (3). Then by a rewetting process, the FS-GFF was attached to the top of separator layer (4).
